# Spectral-Domain Optical Coherence Tomography of the Rodent Eye: Highlighting Layers of the Outer Retina Using Signal Averaging and Comparison with Histology

**DOI:** 10.1371/journal.pone.0096494

**Published:** 2014-05-02

**Authors:** Adeline Berger, Sophie Cavallero, Elisa Dominguez, Peggy Barbe, Manuel Simonutti, José-Alain Sahel, Florian Sennlaub, William Raoul, Michel Paques, Alexis-Pierre Bemelmans

**Affiliations:** 1 Inserm, U 968, Paris, France; 2 UPMC Univ Paris 06, UMR_S 968, Institut de la Vision, Paris, France; 3 Centre Hospitalier National d'Ophtalmologie des Quinze-Vingts, INSERM-DHOS CIC 503, Paris, France; 4 CEA, DSV, I^2^BM, Molecular Imaging Research Center (MIRCen), Fontenay-aux-Roses, France; 5 CNRS, CEA URA 2210, Fontenay-aux-Roses, France; Queen's University Belfast, United Kingdom

## Abstract

Spectral-Domain Optical Coherence Tomography (SD-OCT) is a widely used method to observe retinal layers and follow pathological events in human. Recently, this technique has been adapted for animal imaging. This non-invasive technology brings a cross-sectional visualization of the retina, which permits to observe precisely each layer. There is a clear expansion of the use of this imaging modality in rodents, thus, a precise characterization of the different outer retinal layers observed by SD-OCT is now necessary to make the most of this technology. The identification of the inner strata until the outer nuclear layer has already been clearly established, while the attribution of the layers observed by SD-OCT to the structures corresponding to photoreceptors segments and retinal pigment epithelium is much more questionable. To progress in the understanding of experimental SD-OCT imaging, we developed a method for averaging SD-OCT data to generate a mean image allowing to better delineate layers in the retina of pigmented and albino strains of mice and rats. It allowed us to locate precisely the interface between photoreceptors and retinal pigment epithelium and to identify unambiguously four layers corresponding to the inner and outer parts of photoreceptors segments. We show that the thickness of the various layers can be measured as accurately *in vivo* on SD-OCT images, than post-mortem by a morphometric analysis of histological sections. We applied SD-OCT to different models and demonstrated that it allows analysis of focal or diffuse retinal pathological processes such as mutation-dependant damages or light-driven modification of photoreceptors. Moreover, we report a new method of combined use of SD-OCT and integration to quantify laser-induced choroidal neovascularization. In conclusion, we clearly demonstrated that SD-OCT represents a valuable tool for imaging the rodent retina that is at least as accurate as histology, non-invasive and allows longitudinal follow-up of the same animal.

## Introduction

Rodents are widely used animal models for the study of the retina, both physiologically and during degenerative diseases. The mouse and rat retinal anatomy is indeed similar in many aspects to that of humans. In addition there is a wide variety of mouse strains that reproduce many inherited retinal degeneration observed in humans. *In vivo*, retinal function is usually evaluated by electroretinography [1]. This technique, although providing a reliable functional index, does not allow assessing the actual degeneration of the different neuronal layers in the retina. The most widely used morphological approach in animal experimentation is histology on transverse sections of the retina, on which it is possible to achieve different quantifications and especially the thickness of retinal layers. However, this technique has several drawbacks. First, it is a rather tedious technique. Second, the fixation process can induce artifacts causing contractions and/or expansions of tissue, which then lead to significant measurement variability [2], [3]. Third, this technique is terminal by its nature, so it does not allow longitudinal monitoring of animals. Transparency of ocular tissues has made possible to develop alternative techniques, to directly visualize the retina and longitudinally monitor the degeneration, such as fundoscopy, confocal scanning laser ophthalmoscopy (cSLO) and angiography [4], [5]. Although these techniques allow visualizing the posterior pole of the eye and identifying morphological abnormalities, they do not provide similar data to those obtained by histology on cross-sections of the retina. The only technique that can provide very similar images to transverse histological sections of the retina is spectral-domain optical coherence tomography (SD-OCT) [6]. This method is non-invasive, fast and produces high-resolution cross-sectional images, which are generated by the interference between a reference optical path and another one that is reflected back from the eye [7], [8]. In humans, this technique is currently the gold standard for longitudinal monitoring of retinal degeneration in a large number of pathologies such as age-related macular degeneration [9], [10] and diabetic retinopathy [9], [11] among others. This technique has logically been applied to animal species used in ophthalmic research, and equipments suitable for the rodents' eye examination have been developed. The Bioptigen 840 nm HHP is one of the most used. It provides “*en face*” views of the posterior segment of the eye and scans of transverse sections [12]. These scans are used to measure the various nuclear layers of the retina: inner nuclear layer (INL), outer nuclear layer (ONL) and ganglion cell layer (GCL). However, there are still uncertainties about the histological correspondence of the outermost layers of the retina, i.e. the inner and outer segments of the photoreceptors (PR), the retinal pigment epithelium (RPE) and the choriocapillaris.

In this report, we describe a method for averaging SD-OCT images of the rodent retina that allows a better analysis of fine structures in the outer layers. The higher signal-to-noise ratio of these images allowed us to define in rats and mice interfaces generated by the outer limiting membrane (OLM), the inner segment, the outer segment, the RPE and Bruch's membrane, the choriocapillaris and the large choroidal vessels. In addition, the comparison of pigmented and albino animals underlined the effect of melanin pigment on SD-OCT imaging. The comparison of these results with histological data demonstrates that averaging of SD-OCT images allows thickness quantification of the various retinal layers with greater precision. Finally, we show that the SD-OCT acquisition method we have developed allows to identify discrete events or retinal lesions induced by different types of light insults.

## Material and Methods

### Animals

Six to 12 weeks-old C57BL/6JRj or C57BL/6NRj (with *rd8* mutation) or BALB/cJ mice were purchased from Janvier SA (Le Genest-Saint-Isle, France). *Rhodopsin* ^−/−^ (*rho* ^−*/*−^) mice were provided by Dr Janis Lem (Tufts University, Boston, MA, USA). Mice were maintained at the Institut de la Vision animal facility under pathogen-free conditions. All animals were housed in a 12 h/12 h light/dark cycle with food and water available *ad libitum*.

### Ethics statement

All manipulations were performed in accordance with the association for research in vision and ophthalmology (ARVO) Statement for the Use of Animals in Ophthalmic and Vision Research. In addition, all the experimental procedures were permitted by the Institutional Animal Care and Use Committee, “Comité d'éthique pour l'expérimentation animale Charles Darwin” (ID Ce5/2010/044), which also specifically approved the study reported in the present manuscript.

### SD-OCT imaging

Pupils were dilated with tropicamide (Mydriaticum, Théa, France) and phenylephrin (Néosynephrine, Europhta, France). Animals were then anesthetized by inhalation of Isoflurane (Axience, France) and placed in front of the SD-OCT imaging device (Bioptigen 840 nm HHP; Bioptigen, North Carolina, USA). Eyes were kept moisturized with 9‰ NaCl during the whole procedure. Image acquisitions were performed using the following parameters: Rectangular scan/1000 A-scan per B-scan/100B-scan 1 frame or 4B-scans 16 frames. Acquired images were saved as .avi files and processed with Fiji software (available at http://fiji.sc/Fiji). Firstly, image artifacts due to breathing movements were eliminated by using the StackReg Plugin. Then, using “Z project” function set to “Sum Slices”, each movie was converted into a single image by compiling a Z-projection of all images of the movie. Thus, the final image results from the maximum projection of sixteen or one hundred images sampled every micron, which means 16 or 100 microns retinal width depending on the selected parameter. This manipulation eliminates most of the noise observed on individual images, which helped to show very clearly the reflectance differences present at the level of the outer retina (Fig. S1, Supporting Information S1 and S2). Thickness of retinal layers were manually measured on this maximum projection image in an axis perpendicular to the individual layers and 500 µm from the centre of the optic nerve using Fiji software [13].

#### Subretinal injections

Animals were anesthetized by intraperitoneal injection of ketamine (50 mg/kg, Virbac, France) and xylazine (10 mg/kg, Bayer HealthCare, Germany), and their pupils were dilated as described previously. Subretinal injections were performed with a 33-gauge blunt needle mounted on a 10 µl syringe (Hamilton, USA). Briefly, a hole was created through the sclera/choroid/retina layers using the sharp tip of a 32-gauge needle, and the blunt needle of the Hamilton syringe was then gently inserted into the vitreous through this hole. The needle was then pushed further into the vitreous until crossing the retina to the opposite side of its entry site into the eye. After injection, the needle was left in place for an additional 10 seconds to prevent leakage of the injected fluid. A successful subretinal injection was checked by visualization of subretinal bleb. Mineral oil (Sigma-Aldrich, France) was injected in the subretinal space to visualize boundary between PR and RPE.

### Light-challenge model

This protocol was adapted from our previous study [14]. Briefly, 3-month-old C57BL/6JRj mice were adapted to complete darkness for 12 hours and pupils were daily dilated with 1% Atropin (Novartis, France). Animals were then exposed to green LED light (4500 Lux, JP Vezon équipements, France) for 4 days and subsequently kept in cyclic 12 h/12 h normal animal facility conditions. SD-OCT was performed 3, 7, 14 and 21 days after light exposure.

### Laser-photocoagulation and choroidal neovascularization (CNV) scar quantification

Three-month-old male C57BL/6JRj mice were anesthetized by intraperitoneal injection of ketamine (50 mg/kg) and xylazine (10 mg/kg). Pupils were fully dilated with 1% tropicamide. Coverslips positioned on the mouse cornea were used as a contact lens. Laser-photocoagulations (400 mW, 50 ms, 100 µm spot size) were performed 1 to 2 disc diameters away from the papillae with a Laser Yag 532 Eyelite (Alcon, USA) mounted on a slit lamp (BQ 900, Hagg-Streitt, France). Laser photocoagulation and rupture of Bruch's membrane were confirmed by immediate observation of a bubble. Choroidal neovascularization (CNV) was quantified 7 days after Laser impact and reported as volume units (µm^3^). Volumetric data obtained from SD-OCT sequences, viewed and analyzed with Imaris software (Bitplane, CT, USA) were compared to data obtained by direct measures of oblate spheroid CNV from the same SD-OCT sequences treated by FIJI software as described before. Extrapolated volume was calculated with the following formula (4/3π*a*b^2^)/2 (in which a is the polar radius and corresponds to the measure along the vertical axis and b is the equator radius and corresponds to the horizontal axis). To determine the power of the extrapolation method compared to 3D rendering method, a linear regression was performed with GraphPad software (San Diego, USA).

#### Histology

Animals were euthanized by CO_2_ inhalation. Before enucleation, a mark with an ophthalmic cautery was made at the nasal quadrant of the cornea, so as to subsequently differentiate upper, lower, nasal and temporal quadrants. For Historesin-sections processing, eyes were fixed by immersion in 0.5% glutaraldehyde and 4% paraformaldehyde in PBS for 2 hours, dehydrated by successive ethanol baths, and included in Historesin (Leica Microsystems, Germany). Oriented sections (5 µm thickness) were cut with a microtome (Microm HM 355S, Thermoscientific, USA) and stained with toluidin blue. Slides were scanned with a Nanozoomer 2.0 HT (Hamamatsu, Japan). Each retinal layer was measured manually. For cryostat-sections processing, eyes were fixed in 4% paraformaldehyde in PBS overnight, cryoprotected at 4°C in successive solutions of PBS containing 10, 20 and 30% of sucrose and embedded in a 10% gelatine/30% sucrose solution, before rapid freezing in an isopentane bath cooled to −40°C. Retinal sections (16 µm thickness) were cut with a cryostat (Microm HM560, Thermo Scientific, USA) and stored at −20°C until further use. For nuclear labelling, sections were incubated 5 min with 4,6-diamidino-2-phenylindole (DAPI, Sigma-Aldrich, France), rinsed and mounted under coverslips with Mowiol reagent. ONL thickness was then automatically quantified by a program specially developed on Metamorph software package (Roper Scientific, France), coupled to Nikon Eclipse T*i* inverted microscope.

### Statistics

GraphPad Prism 5 (GraphPad Software, San Diego, USA) was used for data analysis and graphic representation. All values are reported as medians. Statistical analysis is described in legend of each figure. All statistic tests have been implemented with an α-risk of 0.05. Parametric tests have been used after assessment of normal data distribution with the d'Agostino and Pearson omnibus normality test. Statistical significance has been indicated in each figure as following: * = p-value<0.05, ** = p-value<0.01, *** = p-value<0.001, **** = p-value<0.0001.

## Results

### Accuracy and reliability of SD-OCT imaging

**Figure 1:**
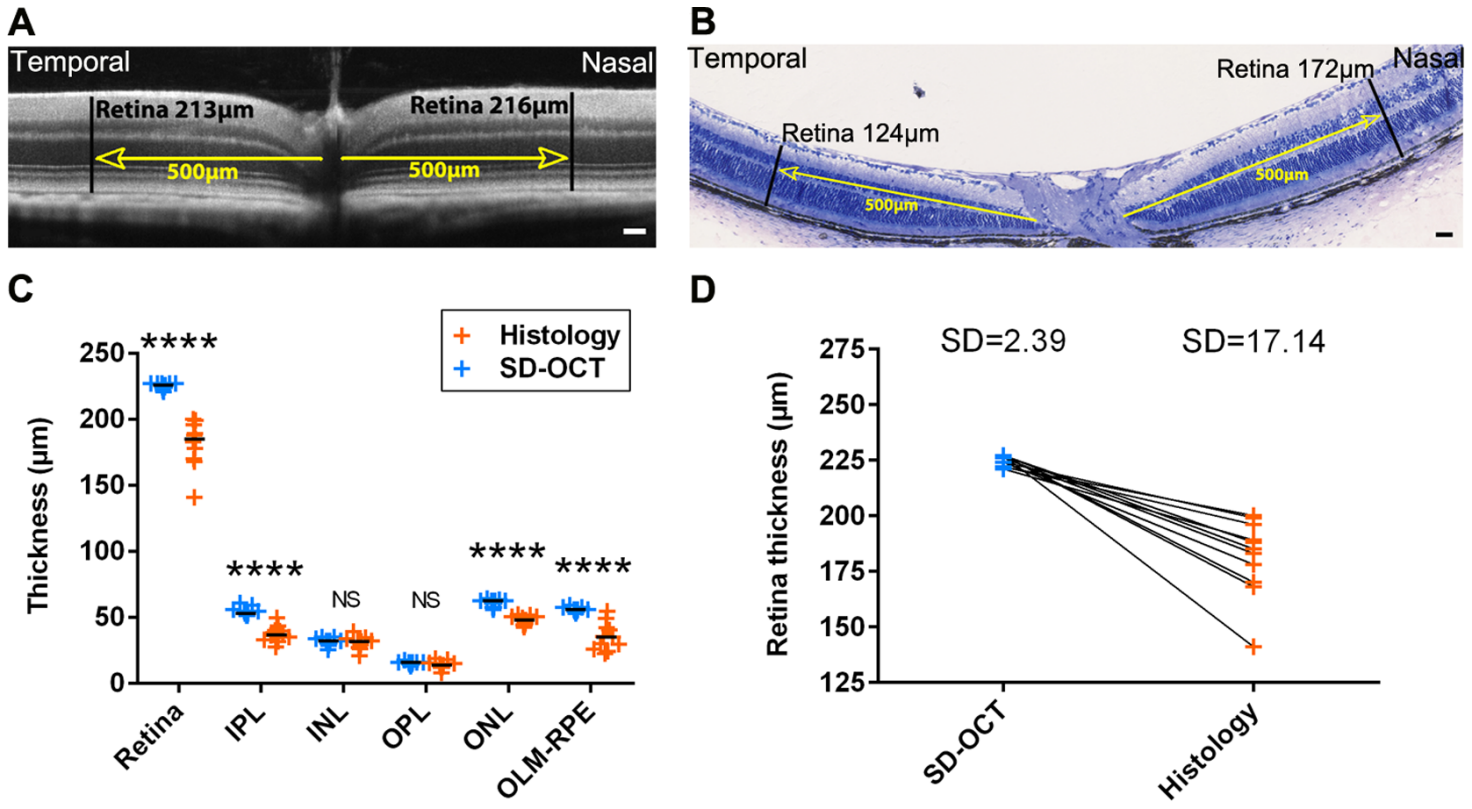
Retinal layer thickness measures in C57BL/6JRj wild-type mice by SD-OCT and histology. Retinal thickness in nasal and temporal sides in SD-OCT image (A) and in corresponding histological section (B). (C) Measures of retinal layers thickness by SD-OCT and histology in C57BL/6JRj mice, n = 11, Mann Whitney test. (D) Retinal thickness evaluated by SD-OCT and histology in C57BL/6JRj mice. Each pair of point represents the whole retina thickness of the same eye measured with SD-OCT (blue dots) and histology (orange dots). IPL: inner plexiform layer, INL: inner nuclear layer, OPL: outer plexiform layer, ONL: outer nuclear layer, OLM: outer limiting membrane, RPE: retinal pigmented epithelium. SD: Standard Deviation. Scale bars: 50 µm.

At first, we wanted to verify that the averaging technique that we used to remove most of the noise from SD-OCT images allowed us to obtain a data quantification that was at least as informative as the one obtained through histological sections. We compared SD-OCT and histological morphometric measures obtained pre- and post-mortem in the same animals. All measures were done at identical locations in SD-OCT images and in corresponding histological sections. As illustrated in Fig. 1A and B, while SD-OCT data showed very similar thicknesses in temporal and nasal sides, more important variations could be observed in histology for the same sample. Moreover, significant differences have been observed between SD-OCT measures and histological ones for the entire retina and some individual layers (Fig. 1C). This could be explained by the treatment of samples for histology. Indeed, we tried to keep our histological samples in good conditions, but it is clear that the extraction and treatments of samples can be responsible of a variation that cannot be excluded in histology and which is preventable with SD-OCT. This phenomenon could also be responsible of the lower reproducibility observed here by histology compared to SD-OCT (Fig. 1D). This highlights that SD-OCT allows a great confidence in data reproducibility simply because *in vivo* measurements avoid potential variations due to sample treatments necessary for *ex vivo* measurements.

### Retinal outer layers characterization in rodents

**Figure 2:**
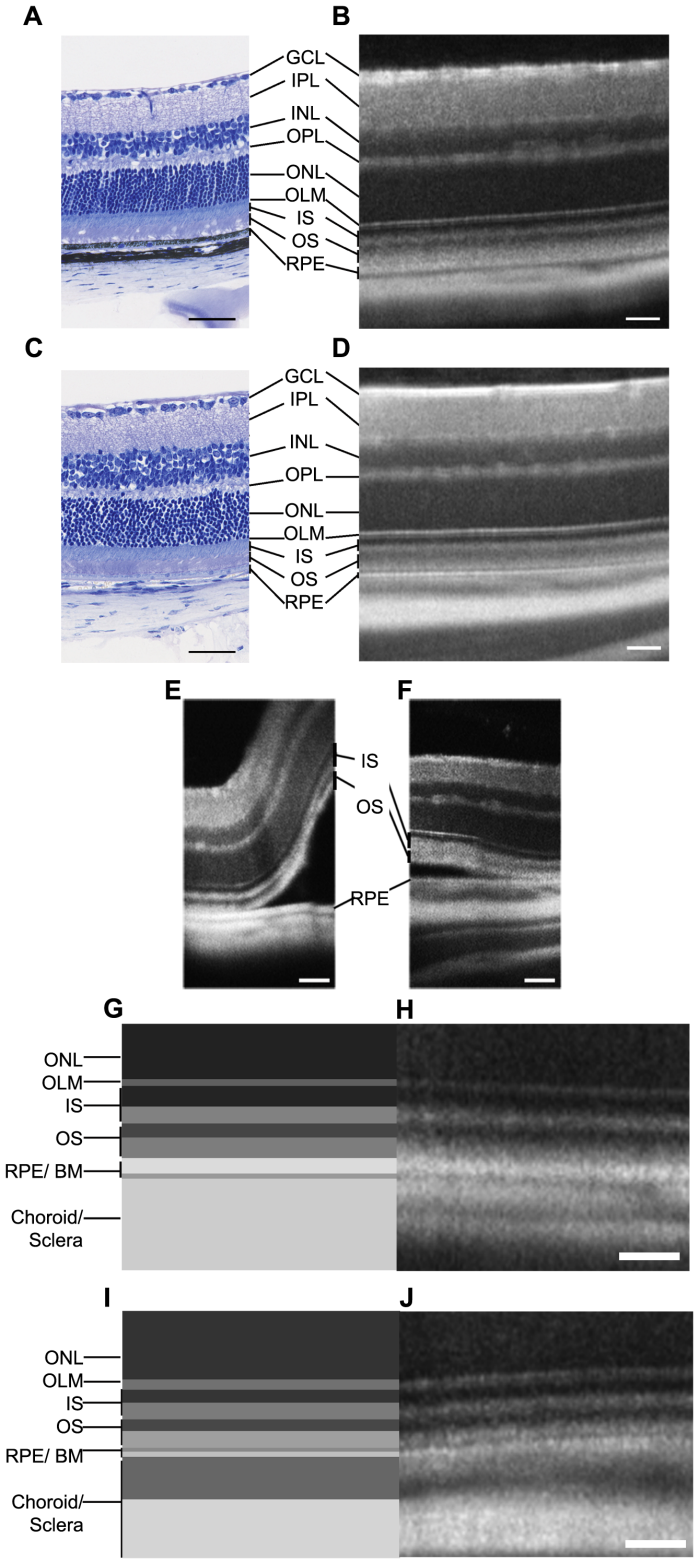
Characterization of pigmented and albino retina layers by SD-OCT. Histological sections of C57BL/6JRj pigmented mouse retina (A) and BALB/cJ albino mouse retina (C). SD-OCT images of pigmented mouse retina (B) and albino mouse retina (D). Retinal detachment induced by subretinal oil injection in C57BL/6JRj pigmented mouse (E) and BALB/cJ albino mouse (F). Schematic representations of pigmented mouse outer retina (G) and albino mouse outer retina (I). Zoom on pigmented mouse outer retina (H) and albino mouse outer retina (J). GCL: ganglion cell layer, IPL: inner plexiform layer, INL: inner nuclear layer, OPL: outer plexiform layer, ONL: outer nuclear layer, OLM: outer limiting membrane, IS: inner segments, OS: outer segments, RPE: retinal pigmented epithelium, BM: Bruch's membrane. Scale bar: 50 µm.

Although SD-OCT allows observation of all retina layers, the outer part of the sensory retina remains less precisely described than the inner one. In order to delimit these outer layers, we compared SD-OCT images to histological sections in pigmented and albino animals. Firstly, we improved the quality of images generated by SD-OCT (Fig. S1) to align layers detected on SD-OCT images and histological sections of the retina (Fig. 2A–D). Comparison of histology and SD-OCT images in pigmented and albino mice revealed the localization of the RPE and showed also notable differences in the SD-OCT imaging of the choriocapillaris (Fig. 2A–D). While in pigmented mice RPE and Bruch's membrane resulted in a white line and a black one from the inner to the outer part of the retina, we observed the opposite in albino mice (Fig. 2B and D), indicating that melanin is a major factor of RPE reflectivity. Also, choroid and sclera appeared clearly defined in albino mice, mainly because of a better light penetration than in pigmented animals in which most of the light is arrested by choroidal pigmentation. To confirm RPE localization, we induced a retinal detachment by subretinal injection of 0.5 µL of oil just before SD-OCT imaging. As expected, detachment occured immediately internal of these above-mentioned retinal layers in pigmented and albino mice (Fig. 2E and F). This line of fractionation induced by the retinal detachment allowed us to precisely define the outer boundary of the PR segments layer, while the inner boundary was itself clearly defined by the outer limiting membrane (OLM), which appeared as a thin hyper-reflective line on the outer edge of the ONL. This allowed us to conclude that the segments of the PR appear as four successive bands of different reflectivity. The first two layers immediately below the OLM presumably correspond to the PR inner segments and the following two layers to the PR outer segments. As expected, no difference could be evidenced at this level between pigmented and albino mice. Based on these results, we can therefore precisely locate the interface between the PR layer and the RPE, and the bands corresponding to the inner and outer portions of the segments (Fig. 2G–J). Analysis of pigmented and albino strains of rats showed similar results (Fig. S2).

### In vivo follow-up in the retina

**Figure 3:**
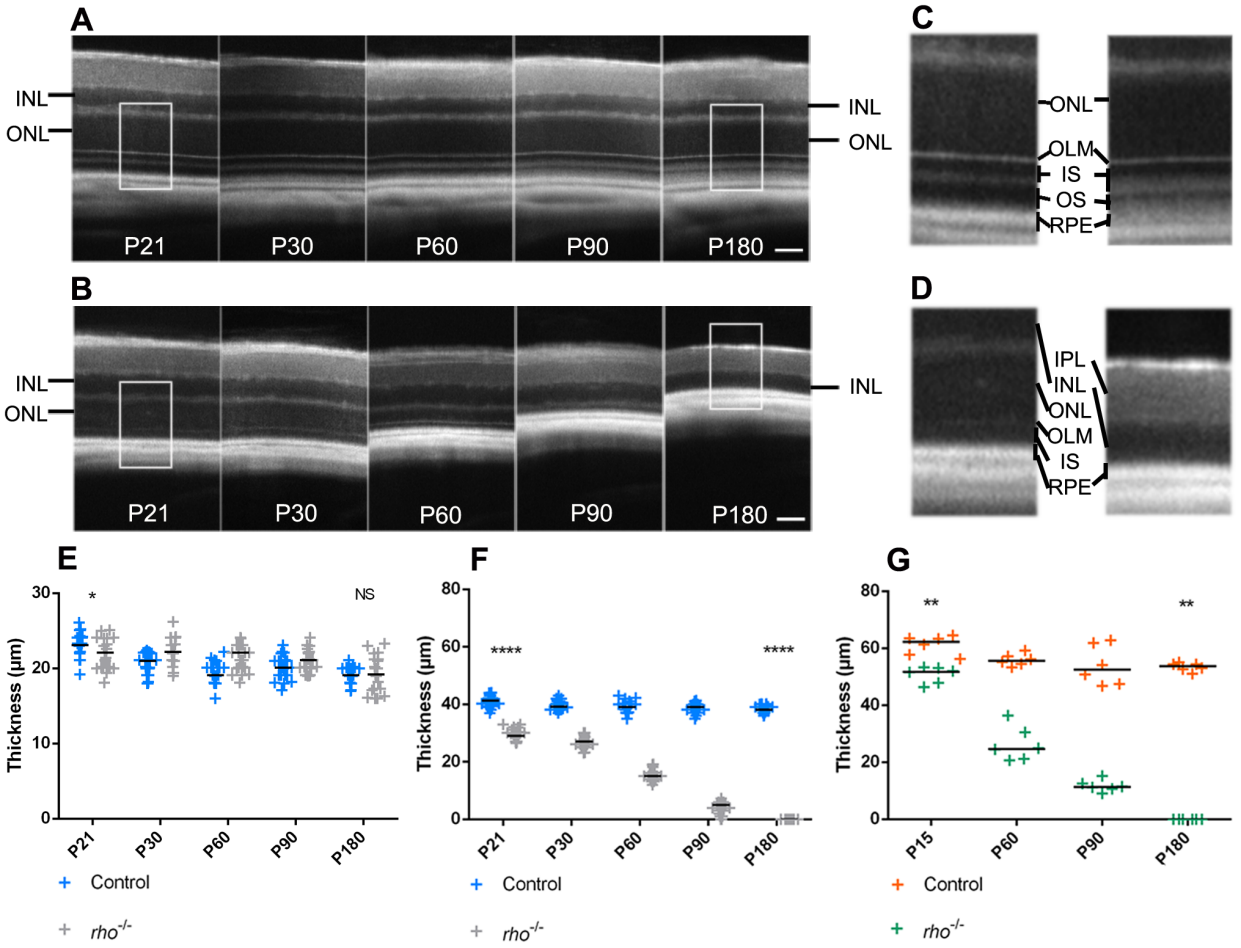
Characterization of a retinal degeneration mouse model by SD-OCT. SD-OCT images of control mice retina (A) and *rho−/−* mice retina (B) from post-natal day 21 (P21) to 180 (P180). Magnification (X2.4) of P21 and P180 control mice outer retina (C) and *rho−/−* mice (D). (E) Measures of INL thickness obtained from SD-OCT data in control and *rho−/−* mice (P21: *p* = 0.0123; P180: *p* = 0.7125). (F) Measures of ONL thickness obtained from SD-OCT data in control and *rho−/−* mice (P21 and P180: *p*<0.0001). (G) Measures of ONL thickness obtained from morphometric measurements on cryostat sections in control and *rho*−/− mice (P15 and P180: p = 0.0022). Statistical significance of the difference between groups was analyzed at the initial time-point (P15 or P21) and the latest time-point (P180) studied by Student's *T*-test for E and F (n = 23 per group) and by Mann Whitney test for G (n = 6 per group). IPL: inner plexiform layer, INL: inner nuclear layer, ONL: outer nuclear layer, OLM: outer limiting membrane, RPE: retinal pigmented epithelium. SD: Standard Deviation. Scale bars: 50 µm.

After having established reliability of SD-OCT for imaging of healthy retina, we wanted to show the usefulness of this technology to characterize the occurrence of physiological or pathological events in the rodent retina. Firstly, we studied degeneration of PR in a *retinitis pigmentosa* mouse model. *Rho^−/−^* mice are a retinal degeneration model [15] that presents a loss of almost all photoreceptors at post-natal day 90 (P90). We followed this degeneration by SD-OCT from P21 to P180, and compared quantification of INL and ONL thickness to those of C57BL/6JRj control mice (Fig. 3A–D). While INL thickness variation was equivalent between *rho^−/−^* and control mice (Fig. 3E), ONL thickness dramatically decreased until complete vanishing at P180 in *rho^−/−^* mice (Fig. 3F). As expected, the same observation was done by histology after DAPI labeling and automated measurements (Fig. 3G). Nevertheless, values' dispersion was far more important with histological measures than with SD-OCT imaging. This highlights that SD-OCT is a valuable tool for *in vivo* monitoring of retinal degeneration in the same animal.

**Figure 4:**
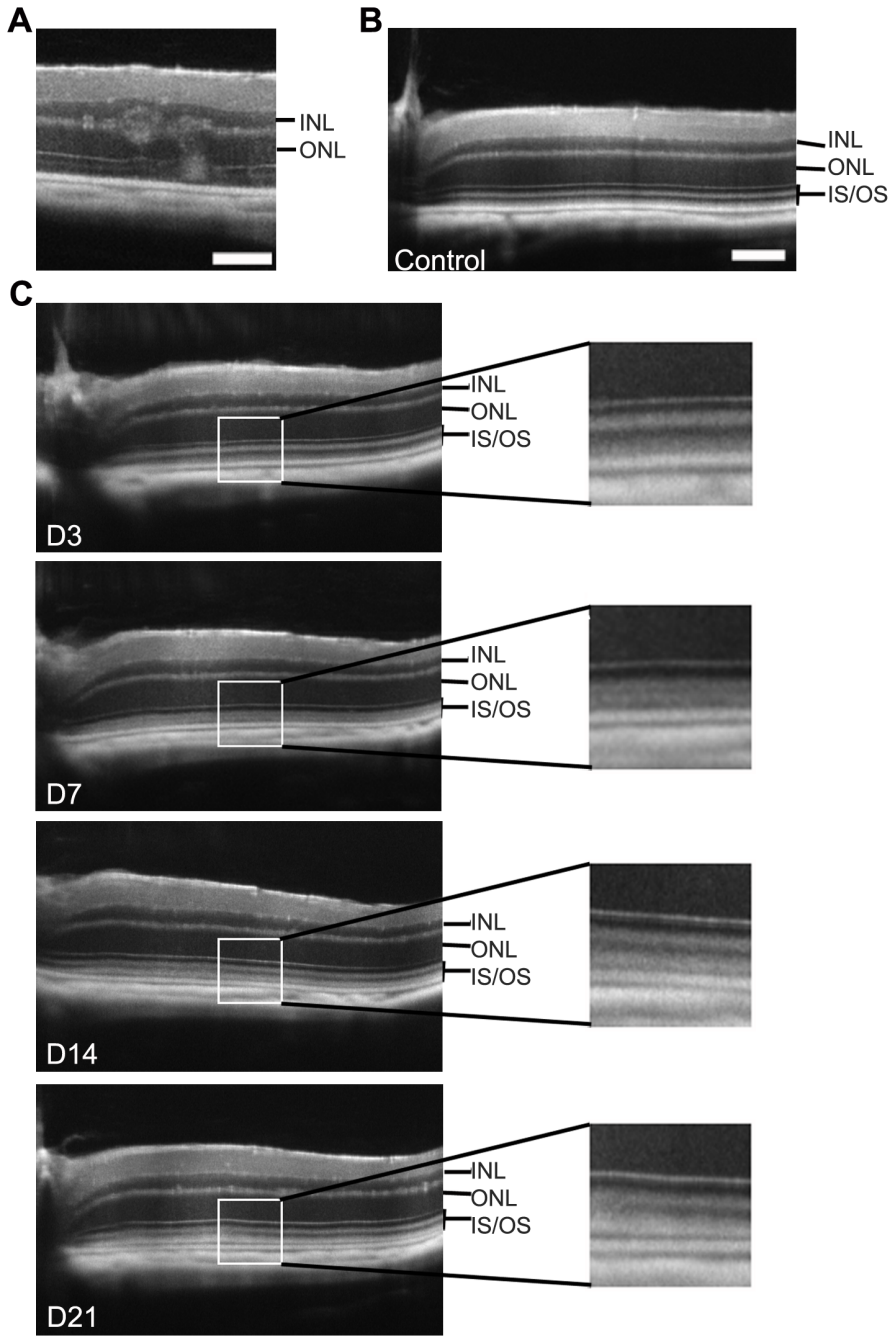
SD-OCT imaging in other pathological models: rd8 mutation and light-challenge. (A) Typical ocular lesions of *rd8* mutation in *crb1* gene (C57BL/6NRj mice in which presence of the *rd8* mutation was confirmed by genotyping). (B–C) SD-OCT follow-up of the outer retina during a light-challenge in C57BL/6JRj mice. Control unexposed three month-old mouse has a normal appearance with 4 bands of different reflectance corresponding to the PR segments (B). Mice were then exposed to light during 4 days as described in the “methods” section and the retina was imaged by SD-OCT at day 3 (D3), 7, 14 and 21 after starting the illumination (C). The light-challenge leads to a temporary abolition of the distinction between the two bands forming the outer segment, with a peak at D7 (right panels: enlargement of the area enclosed by a white box on the left view). INL: Inner Nuclear Layer, ONL: Outer Nuclear Layer, IS: Inner Segments, OS: Outer Segments. Scale bars: 50 µm.

After improving SD-OCT image quality, and following *in vivo* processes, we then questioned whether SD-OCT imaging might also be useful to visualize discrete physiological or pathological events. For example, we wondered if “rosettes” described in C57BL/6NRj carrying the *rd8* mutation in the *crb1* gene [16], were observable by SD-OCT imaging. As shown on Fig. 4A, we indeed easily evidenced these features on *rd8* genotyped mice. Another example is the light-challenge model, a type of light exposition that we developed to study the relationship between oxidative stress, neuroinflammation and photoreceptor apoptosis in different mouse strains [14]. We thus monitored the changes occurring in outer retinal structures during and after exposition to toxic levels of light for 4 days, performed on C57BL/6Rj animals. In albino animals, this type of exposition leads to a massive death of the photoreceptors occurring through an apoptotic mechanism [17]. However, this light regimen, which we call “light-challenge” instead of “light-damage”, does not lead to such PR degeneration in the pigmented animals used here. After 3 days of continuous illumination (Fig. 4C panel D3), SD-OCT imaging showed layers very similar to those of the control non-illuminated (Fig. 4B) even if the white layer of outer segments seemed to disappear. At day 7 (i.e. 3 days after stopping continuous illumination) the two different layers defining the outer segments were no longer distinguishable (Fig. 4C panel D7 and Fig. S3). This phenomenon was just temporary, because at D14 and D21, inner and outer segments had regained their original appearance. One of the hypotheses to explain this variation is a possible link with inflammatory processes already described [14]. This observation further confirmed the usefulness of SD-OCT imaging to monitor *in vivo* the occurrence of discrete events in the rodent retina.

**Figure 5:**
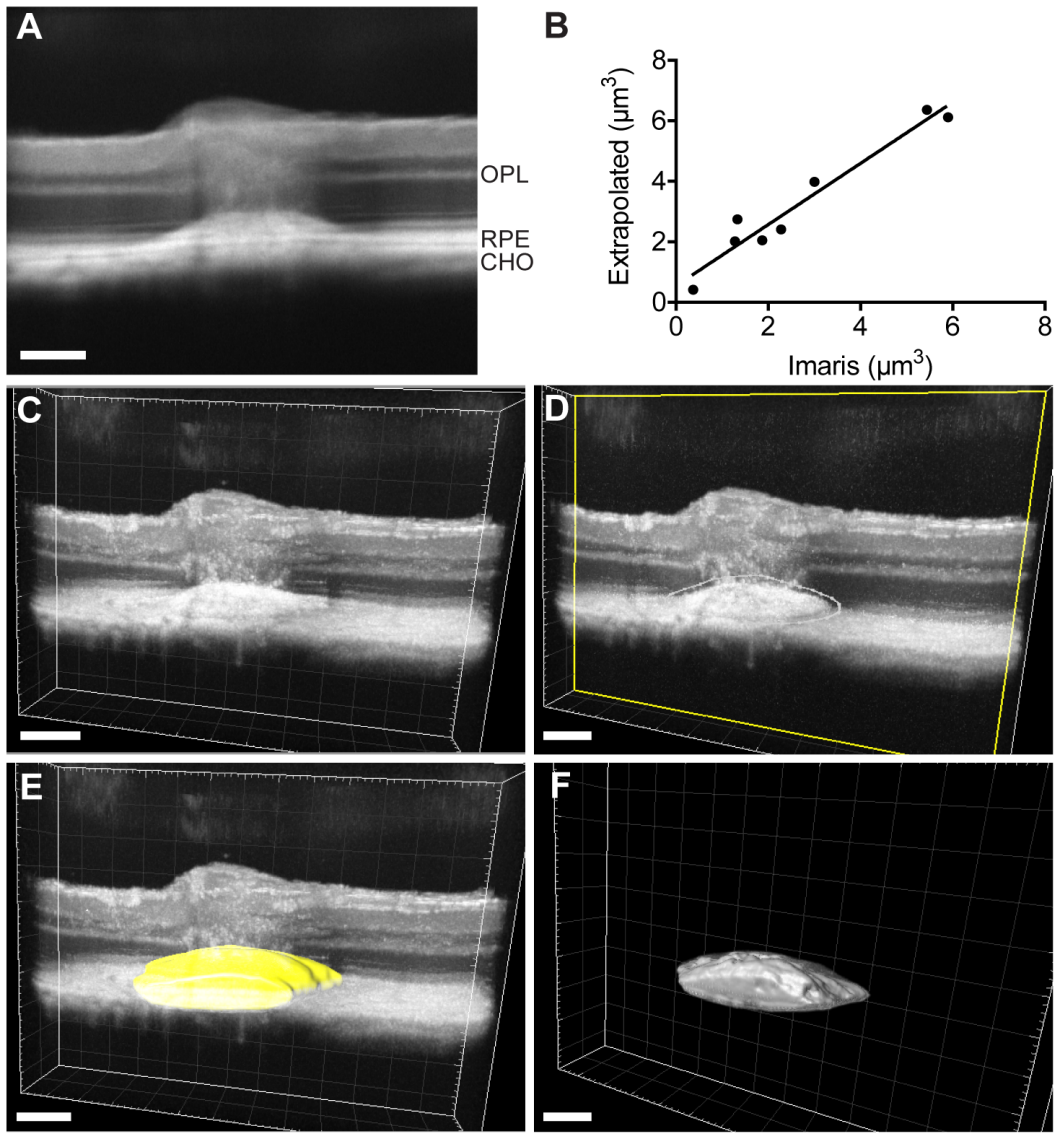
Quantification of laser-induced choroidal neovascularization (CNV) in C57BL6/JRj. (A) Laser-induced CNV (Yag 532 Eyelite parameters: 100 µm, 50 ms, 400 mW) was visualized immediately after laser impact using SD-OCT imaging as described in the “materials and methods” section. Based on this image, a CNV volume is extrapolated using the following formula (4/3π*a*b^2^)/2, in which *a* is the polar radius and corresponds to the measure along the vertical axis and *b* is the equator radius and corresponds to the horizontal axis. (B) Linear regression showing that data obtained from extrapolation or Imaris 3D reconstruction (described step by step hereafter) are statistically equivalent (r^2^ = 0,94, n = 8). (C) Imaris software allows a 3D rendering of SD-OCT imaging. Data shown here arise from the same SD-OCT sequence than shown in panel A. (D) The neovascularization volume, just above the RPE cell layer, was delimitating manually (representative white dotted line in one slice) in about 20 slices (over 100) along z-axis to create a 3D mask. Based on this manual delimitation the Imaris software computed a 3D mask shown in yellow (E). The final visualization, that allowed CNV volume quantification, was obtained after automated mask thresholding (F). OPL: Outer Plexiform Layer, RPE: Retinal Pigmented Epithelium, CHO: Choroid. Scale bar: 50 µm.

Finally, SD-OCT imaging can also provide detailed structure of choroidal neovascularization (CNV) occurring above the RPE (Fig. 5A) after laser photocoagulation. Laser-induced CNV indeed mimics some major aspects of wet age-related macular degeneration [18]. We wondered whether extrapolated CNV volumes obtained by direct quantification of SD-OCT images (after signal integration as described in the methods section) were comparable with volumetric data obtained from the same SD-OCT sequences viewed and analyzed with Imaris software (Fig. 5C–F). Here we showed that direct measures on SD-OCT sequences represented a faster and reliable alternative to CNV analysis using a dedicated 3D rendering software (Fig. 5B). We sought here to provide a new procedure to optimize SD-OCT imaging and CNV quantification. All together these data show that SD-OCT imaging is a powerful tool to describe different phenomena affecting the retina and not only reduction of layers thickness.

## Discussion

During the last decade, SD-OCT imaging has taken a prominent place in the clinic for the follow-up of patients with retinal degeneration [19]. Application of this technology to rodents has been developed more recently, in part probably because in first intention histological tissue analysis seems more informative and versatile. In this study, we aimed at investigating the specific interest of SD-OCT for the study of rodent models of retinal degeneration. We first developed a method for acquisition and averaging of images that allowed to increase the signal to noise ratio. With this method we could analyze on SD-OCT images the nature of the different reflectance layers of the outer retina of pigmented and albino strains of rodents. Comparison of the results obtained after histological analysis or quantification of SD-OCT imaging finally clearly demonstrated the accuracy and reproducibility of the latter for the quantification of retinal degeneration in rodents. This was certainly due to tissue shrinkage or expansion occurring during histological processing that was not consistent from one sample to another or even within a given sample. Fixation itself is the source of artifacts [3]. Thus, in our study, we observed a tendency to tissue retraction with Historesin embedding and microtome sectioning (Fig. 1), and rather a trend to dilation following gelatin/sucrose embedding and cryostat sectioning (Fig. 3). Similarly, Jiao and colleagues showed in a recent study on rats that retinas processed by paraffin embedding displayed a significant tissue retraction compared with those treated by cryostat sectioning [20]. This clearly highlights one of the advantages of SD-OCT imaging, which is to collect *in vivo* data, allowing to monitor over time the same animal, and avoiding potential variations due to sample treatments.

Interpretation of reflectance variations of the outermost retinal and choroidal layers remains debated [21], [22]. This is due to the uncertainty that exists as to the exact boundary between the RPE and PR and also the relatively noisy appearance of SD-OCT images in rodents. In addition, the number of layers detected between OLM and RPE through SD-OCT imaging (4 according to our interpretation described below) do not match the number of layers observed in histology (2, corresponding to the inner and outer part of PR segments). We circumvented the first hurdle by implementing an averaging of the acquisitions, which significantly increased the signal-to-noise ratio compared to conventional rodent SD-OCT images obtained with the Bioptigen 840 nm HHP [23], [24]. We then used an experimental artifice consisting in detaching the neuroretina from the RPE by subretinal injection of oil, to precisely locate the interface between the RPE and PR. This allowed us to confirm the location of the inner segment and outer segment (Fig. 2G and H) each corresponding to two bands of different reflectance. This interpretation is consistent with that recently proposed by Spaide and Curcio for the human peripheral retina, obtained after comparison of histological findings from dozens of publications with SD-OCT data [25]. For the inner segment, the two bands most likely match to myeloid and ellipsoid [26]. For the outer segment, the presence of two bands can be explained by the microvilli of the RPE ensheathing only the most peripheral part of the outer segment of the PR. In humans, these microvilli can indeed extend for as long as half of the outer segment [27]. The RPE and the PR layers are thus entangled into one another, which may explain at least partly the poor contrast observed by SD-OCT between these two layers. In contrast, the basal pole of the RPE and the Bruch's membrane appear perfectly delineated. External to this structure, the presence of three layers of different reflectance most likely corresponds to the choriocapillaris, choroid and sclera layers (Fig. 2G–J), although there is no definitive clue allowing to clearly establish the boundaries of these structures. Finally, the comparison between albino and pigmented animals shows as expected that no reflectance difference is observed in these two genetic backgrounds for the non-pigmented cells layers. However, peripheral from the RPE, we observed a greater light penetration and less defined boundaries in albino animals (Fig. 2 and Fig. S2). In particular, the OCT imaging of the choroid showed notable variations linked to the presence of melanin: while whole choroidal thickness was better imaged in albino eyes, the choroicapillaris was distinguishable only in pigmented eyes.

Finally, a significant advantage of SD-OCT imaging compared to conventional histological techniques when considering ethical issues, is the ability to perform more easily longitudinal follow-up of animal models. For example, for the study of the time-course of retinal degeneration in *rho−/−* mice, a total of only 12 animals is in theory necessary for acquisition of SD-OCT data (n = 6 per group), whereas 48 animals are needed for histology data (n = 6 per group). Thus, the use of SD-OCT imaging allows a very significant gain in the amount of work needed to achieve a more reliable result. Moreover, this technique is applicable to a large number of pathological situations. It allows not only to detect subtle variations in retinal layers thickness, reflecting a possible degeneration, but also to highlight other phenomena, such as the change in reflectance of the segments layers observed after exposure of the animals to light-challenge, even with moderate levels of light. Presumably an acceleration of phagocytosis of photoreceptors discs and/or a phenomenon of photostasis associated with inflammatory processes are causing these changes in reflectance. In the CNV model, our goal was not to compare benefits obtained from histological findings or SD-OCT images, which have been previously discussed. Previous studies [20], [28], [29] showed that CNV size or thickness follow-up using SD-OCT was comparable with data obtained from histological sections. Here we sought to provide a non-invasive and optimized tool to quantify CNV. Based on integrated SD-OCT images, we propose a simple method of calculation that allows a fast and reliable CNV quantification comparable with 3D rendering. Thus, SD-OCT imaging can not only accurately quantify retinal layers thickness, but also highlight more subtle phenomena reflecting metabolic changes in the tissue. However, the histology remains an indispensable complement to the study of animal models due to the versatility of the techniques that can be implemented (histochemical staining, immunolabeling, in situ hybridization, etc.) and the fact that histology gives access to a cellular resolution allowing for example to quantify the number of rows of PR's nuclei in the ONL. In addition histology is useful when the peripheral retina is particularly affected, because this part of the retina is barely accessible to SD-OCT imaging.

In conclusion, signal averaging of SD-OCT scans in rodent increases its capacity to quantify the thickness of retinal layers with a strong reproducibility. Moreover, it allows a better determination of reflectance's correlation with anatomical structures of the outer retina layers. All these features make of SD-OCT an ideal tool for the exploration of various rodent pathological models that can be followed-up longitudinally in the same eye.

## Supporting Information

Figure S1 **Enhancement of SD-OCT resolution by image averaging.** The fundus of a C57BL/6JRj mouse was imaged by SD-OCT using the Bioptigen 840 nm HHP device. Individual scans are relatively noisy and do not allow to precisely delineate the different layers of the outer retina located peripheral to the outer limiting membrane (A). After acquisition and averaging by the ImageJ software of 16 images separated from each other by 1 µm, these layers appear much more clearly (B). Scale bar  = 50 µm.
(TIF)

Figure S2 **Comparison of SD-OCT images and histological sections of pigmented and albino rat retina.** Histological sections of Long Evans pigmented rat retina (A) and Wistar albino rat retina (D). SD-OCT images of pigmented rat retina (B) and albino rat retina (C). Zoom on pigmented rat outer retina (E) and albino rat outer retina (F). GCL = Ganglion Cell Layer, IPL = Inner Plexiform Layer, INL = Inner Nuclear Layer, OPL = Outer Plexiform Layer, ONL = Outer Nuclear Layer, OLM = Outer Limiting Membrane, IS = Inner Segments, OS = Outer Segments, RPE = Retinal Pigmented Epithelium, Bruch M = Bruch Membrane. Scale bar  = 50 µm.
(TIF)

Figure S3 **Eyes of 3 animals at day 7 of light-challenge.** Right and left panels represent respectively right and left eyes of 3 C57BL/6JRj mice at day 7 after starting of light-challenge (i.e. 3 days after stopping continuous illumination). Scale bar  = 50 µm.
(TIF)

Supporting Information S1 **SD-OCT.avi files before processing with ImageJ.**
(AVI)

Supporting Information S2 **SD-OCT.avi files after processing with ImageJ.**
(AVI)
