# Supplementary figures and images for: Spectral-Domain Optical Coherence Tomography of the Rodent Eye: Highlighting Layers of the Outer Retina Using Signal Averaging and Comparison with Histology
Source: PLoS One. 2014 May 2;9(5):e96494. doi: 10.1371/journal.pone.0096494 (PMC4008571; doi:10.1371/journal.pone.0096494)

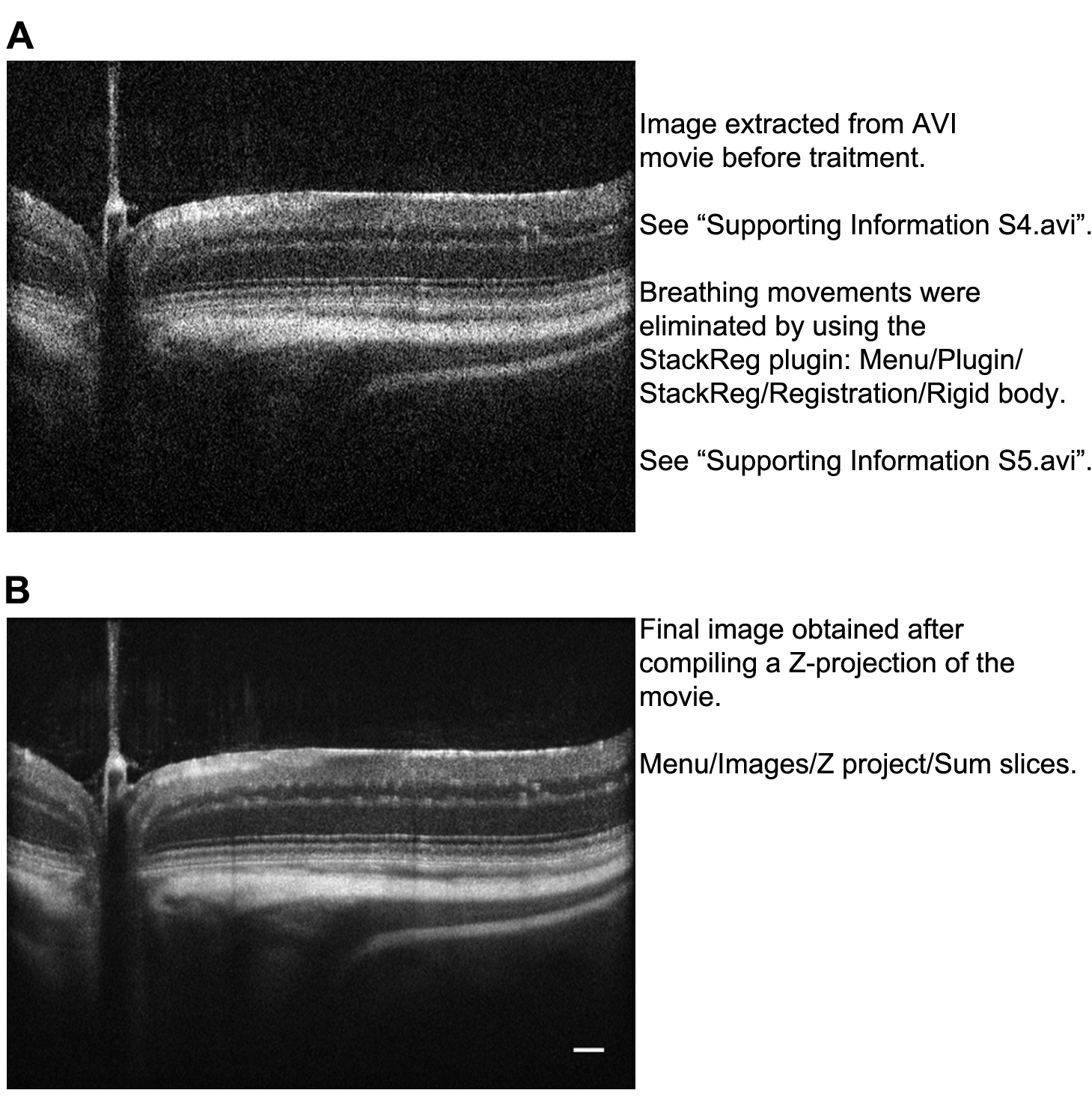

Supplement: Figure S1 — Enhancement of SD-OCT resolution by image averaging. The fundus of a C57BL/6JRj mouse was imaged by SD-OCT using the Bioptigen 840 nm HHP device. Individual scans are relatively noisy and do not allow to precisely delineate the different layers of the outer retina located peripheral to the outer limiting membrane (A). After acquisition and averaging by the ImageJ software of 16 images separated from each other by 1 µm, these layers appear much more clearly (B). Scale bar = 50 µm. (TIF) [file pone.0096494.s001.tif]

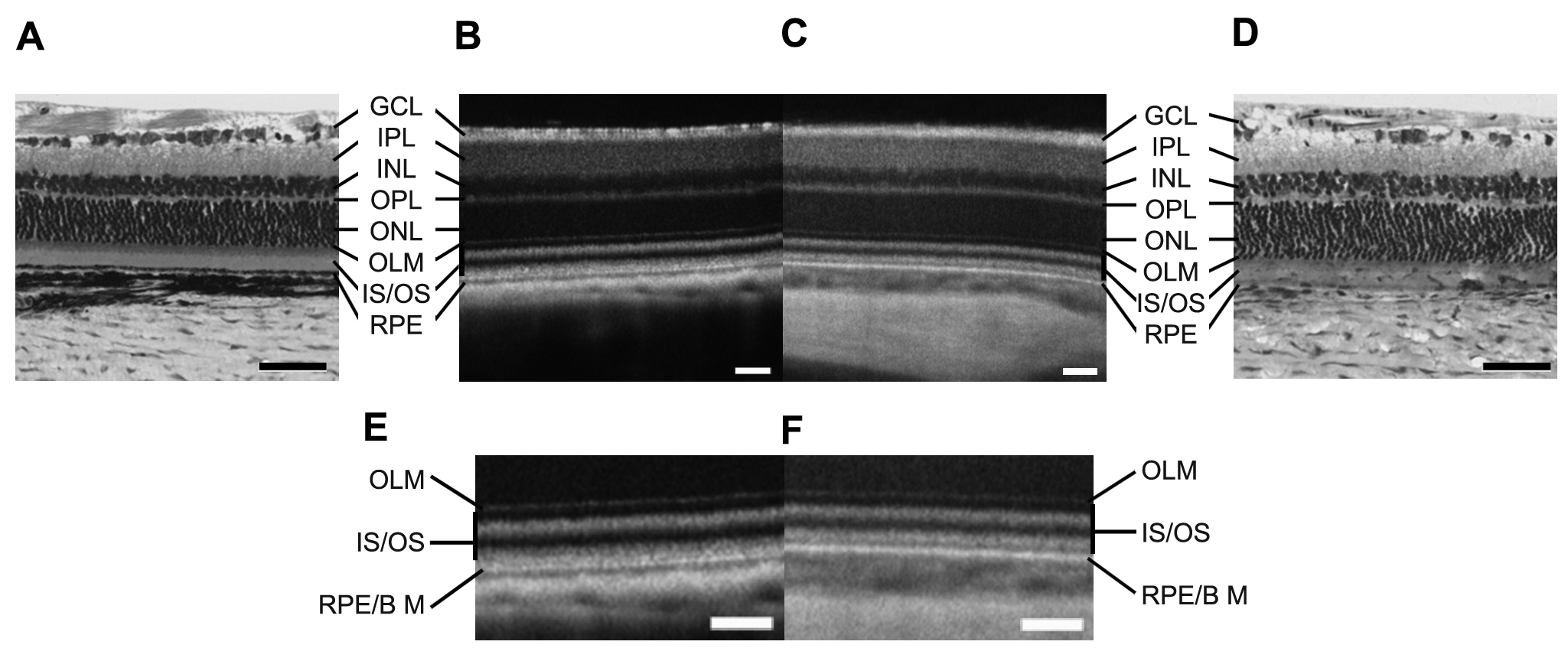

Supplement: Figure S2 — Comparison of SD-OCT images and histological sections of pigmented and albino rat retina. Histological sections of Long Evans pigmented rat retina (A) and Wistar albino rat retina (D). SD-OCT images of pigmented rat retina (B) and albino rat retina (C). Zoom on pigmented rat outer retina (E) and albino rat outer retina (F). GCL = Ganglion Cell Layer, IPL = Inner Plexiform Layer, INL = Inner Nuclear Layer, OPL = Outer Plexiform Layer, ONL = Outer Nuclear Layer, OLM = Outer Limiting Membrane, IS = Inner Segments, OS = Outer Segments, RPE = Retinal Pigmented Epithelium, Bruch M = Bruch Membrane. Scale bar = 50 µm. (TIF) [file pone.0096494.s002.tif]

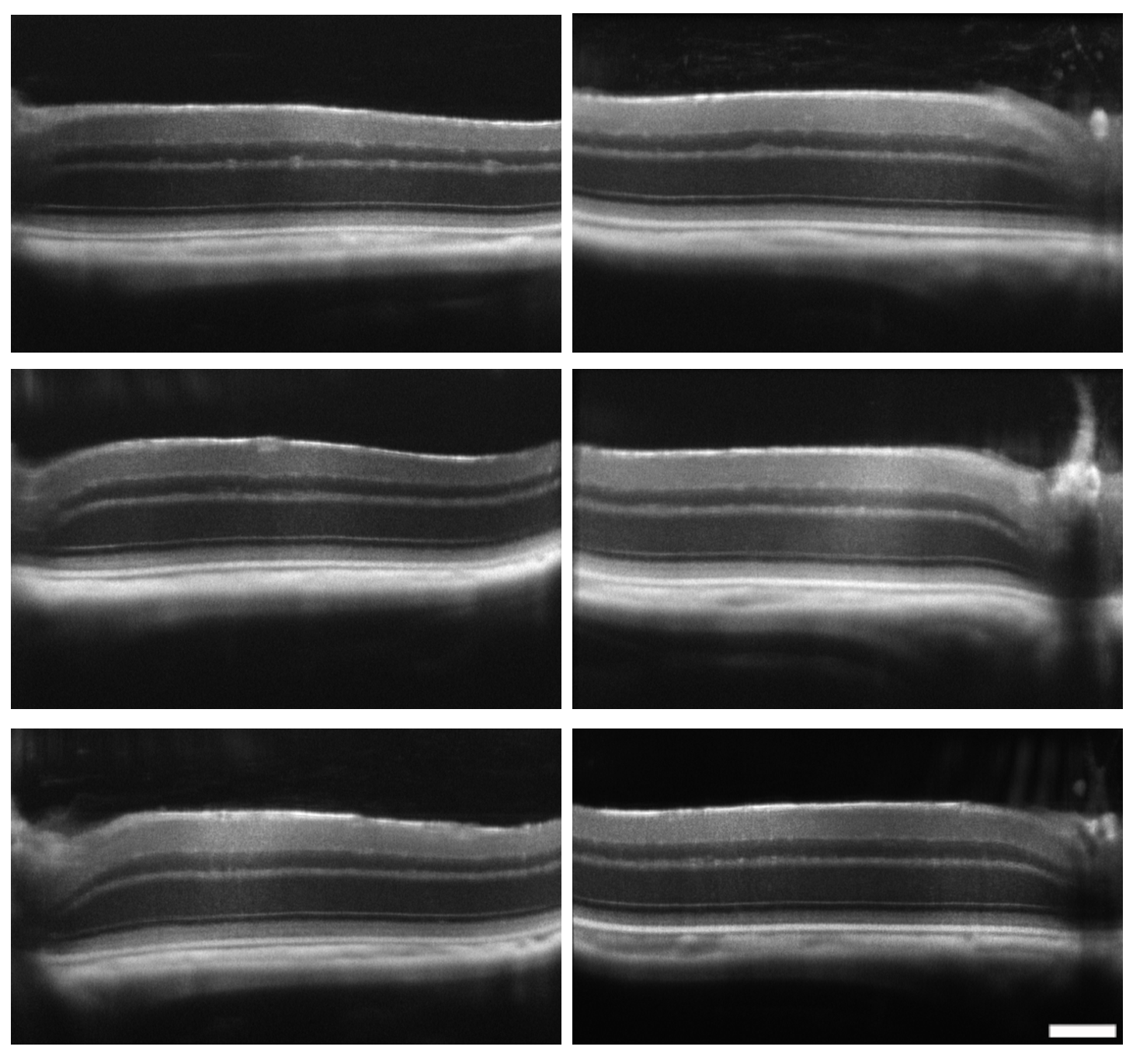

Supplement: Figure S3 — Eyes of 3 animals at day 7 of light-challenge. Right and left panels represent respectively right and left eyes of 3 C57BL/6JRj mice at day 7 after starting of light-challenge (i.e. 3 days after stopping continuous illumination). Scale bar = 50 µm. (TIF) [file pone.0096494.s003.tif]
